# Point of Care Contrast Enhanced Ultrasound Utility in the Diagnosis of a Gallbladder Perforation: A Case Report

**DOI:** 10.24908/pocusj.v10i01.18166

**Published:** 2025-04-15

**Authors:** Lauren Lu, Rebecca Leff, Tobias Kummer

**Affiliations:** 1 Department of Emergency Medicine, Detroit Receiving Hospital, Wayne State University, Detroit, MI, USA; 2 Division of Emergency Medicine, Boston Children's Hospital, Harvard Medical School, Boston, MA, USA; 3 Department of Emergency Medicine, Mayo Clinic, Rochester, MN, USA

**Keywords:** Contrast-enhanced ultrasound, acute cholecystitis, biliary peritonitis, gallbladder perforation

## Abstract

We detail the case of a 61-year-old patient with a history of metastatic ovarian cancer who presented to our emergency department (ED) with findings consistent with localized gallbladder perforation and abscess development on an outpatient computed tomography (CT) scan. The differential etiology included primary cholecystitis or infiltrative metastatic mass in the setting of known peritoneal carcinomatosis. Point of care contrast-enhanced ultrasound (CEUS) examination was performed and was able to rule out local tumor infiltration, which was later confirmed by pathology after cholecystectomy. In this case, the identification and confirmation of gallbladder perforation on imaging allowed for more conservative management with image-guided drain placement in a patient who was not an optimal surgical candidate at the time and underwent delayed cholecystectomy. This case report emphasizes the role of CEUS in providing detailed information about gallbladder wall perfusion and integrity with the potential to predict impending gallbladder perforation by demonstrating areas of gallbladder wall hypoperfusion and necrosis as well as visualizing infiltrative lesions.

## Introduction

Spontaneous perforation of the gallbladder is a rare disease with reported mortality rates ranging from 12-42% and a high complication rate requiring urgent intervention [1]. As signs and symptoms of gallbladder perforation are comprised of a wide range of clinical findings and do not differ significantly from those of uncomplicated cholecystitis, the diagnosis is often delayed or misdiagnosed, leading to increased mortality [1]. Risk factors for spontaneous gallbladder perforation include cholelithiasis, infection, intra-abdominal malignancy, diabetes, and steroid therapy [2]. Perforation most commonly occurs at the gallbladder fundus because of its limited blood supply [3]. Given the diagnostic difficulties based on the clinical history and physical examination, diagnosis of this condition has traditionally been reliant on laboratory findings, diagnostic computed tomography (CT), and ultrasound (US) imaging [4, 5]. However, the accurate and timely diagnosis of this condition remains challenging given the poor sensitivity of traditional imaging modalities and is often only confirmed by direct intraoperative visualization or postoperative pathology [6, 7].

The difficulty of this diagnosis with traditional CT and US imaging has been highlighted in several studies. In a comparative study of CT and US for the diagnosis of gallbladder perforation in 13 patients, Bennet et al. detected the site of perforation in only 50% of patients on CT and in no patients using ultrasonography [8]. Similarly, Kim et al. identified a gallbladder wall defect or bulging of the gallbladder wall, suggesting a site of perforation in only 38.5% of patients studied (n=5) using US and 69.2% of patients studied (n=9) using CT [9]. Two studies evaluating a total of 29 patients found poor US sensitivity in patients diagnosed intraoperatively with gallbladder perforation, and retrospective reviewers were unable to find evidence of gallbladder perforation on US imaging [7, 8]. B-mode and color Doppler US are the most commonly used modalities to diagnose gallbladder pathology in the emergency department (ED), with sensitivities ranging from 67.1-89.9% for cholecystitis [10, 11]. However, when gallbladder disease is complicated by lumen-altering pathology such as a gallbladder wall defect, limitations arise in the usage of diagnostic B-mode and color Doppler US [12, 13].

We present the case of a 61-year-old woman with gallbladder perforation diagnosed by point of care contrast-enhanced ultrasound (CEUS). CEUS utilizes microbubble contrast agents to provide valuable information of tissue perfusion at a far greater level than conventional color or spectral Doppler US imaging, with a role more analogous to contrast-enhanced CT or medical resonance imaging (MRI) [14]. Ultrasound contrast agents (UCAs) are composed of microbubble gas cores encapsulated by a lipid or protein shell. As the microbubbles used in CEUS are similar in size and rheologic properties to erythrocytes, they remain confined to the intravascular space. This makes CEUS an effective modality for detecting tissue perfusion [15]. Presently, CEUS is most useful in situations where dynamic real-time acquisition of abdominal imaging perfusion data is of value, especially in the case of severely ill patients for whom transport to CT imaging is challenging or for whom renal insufficiency is a relative contraindication to CT contrast imaging [16,17]. The challenging and uncommon diagnosis of gallbladder perforation is often delayed or missed, contributing to increased morbidity and mortality. In some of these cases, the use of CEUS may improve the diagnostic accuracy of US, leading to more rapid diagnosis by allowing for evaluation of gallbladder wall integrity [18].

## Case report

**Figure 1.**
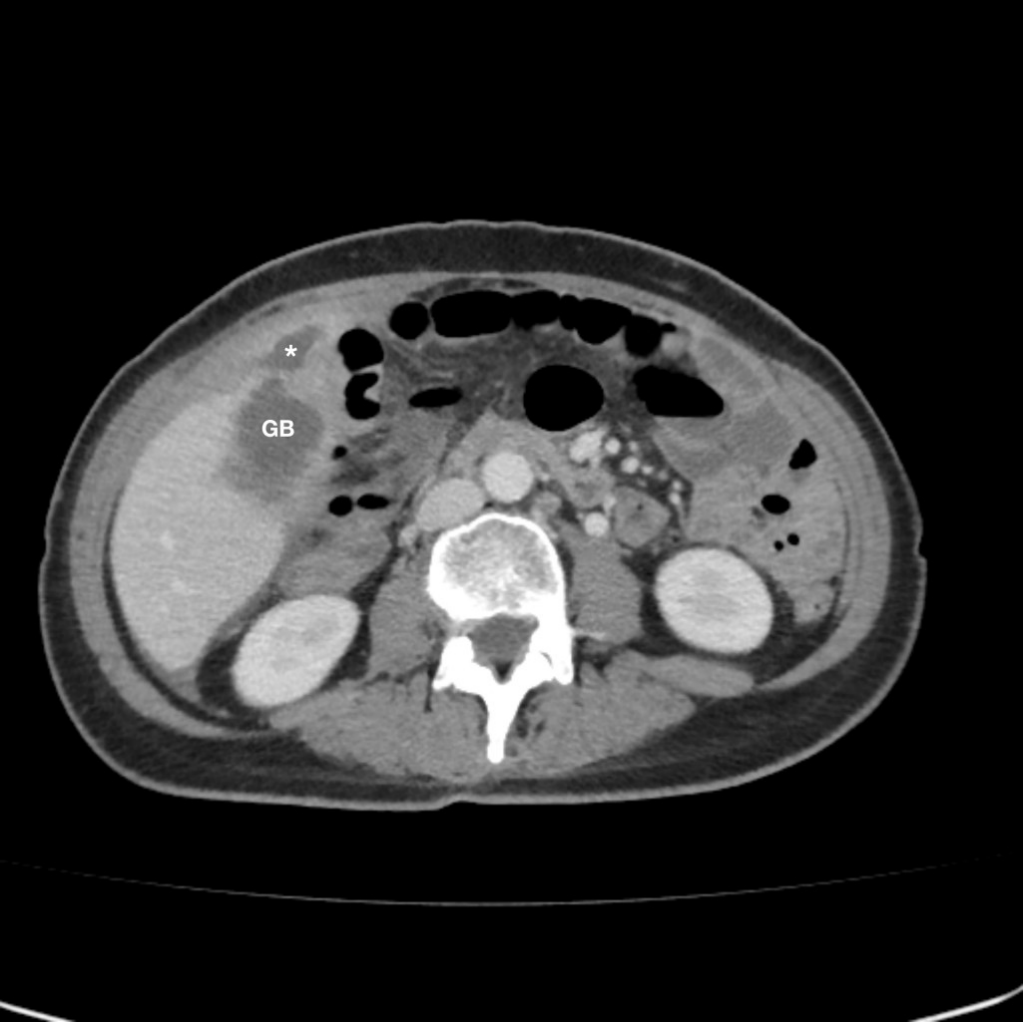
CT scan. The computed tomography (CT) scan shows a distended gallbladder with a thickened enhancing nodular wall and an adjacent fluid collection (*) concerning for gallbladder perforation.

A 61-year-old woman with metastatic ovarian cancer presented to the ED with evidence of localized gallbladder perforation with abscess development on an outpatient CT scan obtained as part of a routine consultation for her ovarian cancer. CT abdomen/pelvis with intravenous contrast revealed thickened nodular enhancement on the wall of the gallbladder with evidence of perforation and a large abscess extending into the right paracolic gutter and anterior pelvis. It was unknown if these findings were due to primary cholecystitis or tumor infiltration with secondary rupture in the setting of known carcinomatosis (Figure 1). She had a history of malignant ascites and pleural effusions requiring multiple prior para- and thoracenteses, bowel obstruction, and choledocholithiasis with sphincterotomy and stone removal two months prior. She had received three cycles of carboplatin and paclitaxel, with the most recent cycle concluding the day prior to presentation to the ED.

**Figure 2.**
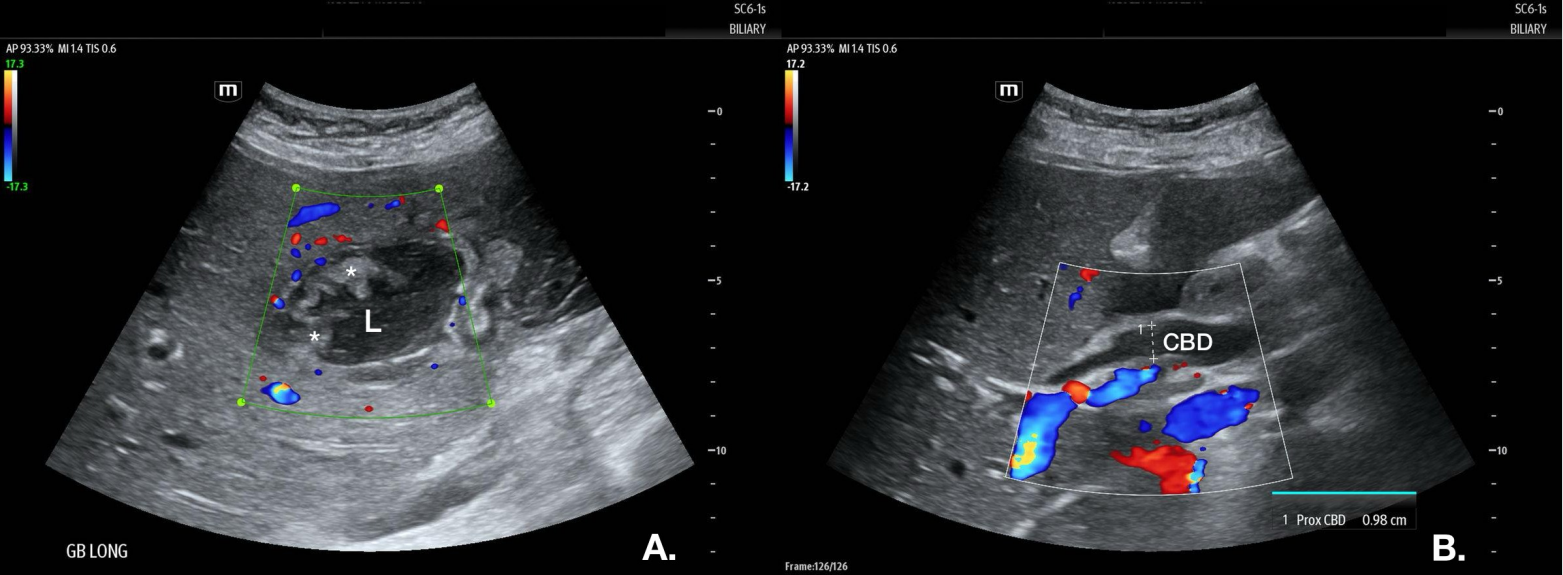
B-mode Ultrasound. B-mode ultrasound (US) with color Doppler overlay of the gallbladder. (Figure 2a) shows hyperechoic echoes (*) in the gallbladder lumen (L) and gallbladder wall thickening with associated hyperemia (red/blue color). The proximal common bile duct is dilated to 0.98 cm (Figure 2b).

**Figure 3.**
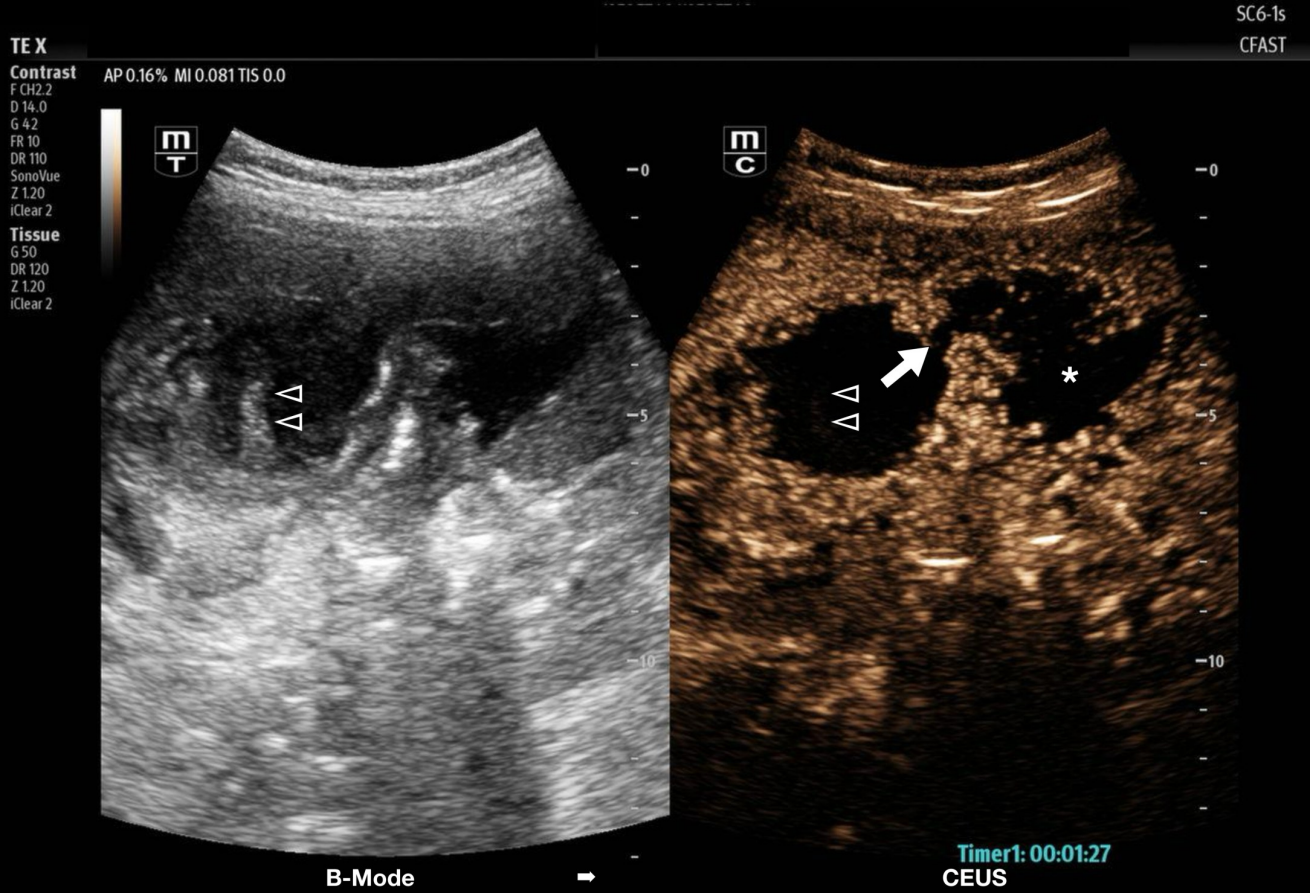
Contrast-enhanced ultrasound B-mode reference image is shown on the left (B-mode), and contrast-enhanced ultrasound (CEUS) image on the right. The CEUS image demonstrates perforation of the gallbladder wall (white arrow) with an adjacent non-enhancing fluid collection. The internal luminal echoes seen on B-mode ultrasound (US) (triangles) are unenhanced consistent with debris in the lumen.

On presentation, she was asymptomatic, reporting no pain, fever, nausea, or vomiting increased from her baseline. She was afebrile (37.2°C), with a respiratory rate of 18, heart rate of 116 per minute, and blood pressure of 102/63mmHg. Laboratory work-up showed no leukocytosis or elevated bilirubin but an elevated ESR of 102 and CRP of 119. Her abdomen was soft and non-distended with right-sided guarding; however, she denied tenderness with palpation and had a negative Murphy's sign. Standard B-mode point of care US using a C6-1s curvilinear transducer showed marked, irregular gallbladder wall thickening with hyperemia on color Doppler and hyperechoic luminal echoes, but no gallstones or classic pericholecystic fluid (Figure 2a). The sonographic Murphy's sign was negative, and the proximal common bile duct was dilated to 0.98 cm (Figure 2b). Given the diagnostic uncertainty of the CT scan and the finding of hyperechoic echos vs. mass in the gallbladder lumen, we decided to proceed with the contrast US study using the same transducer as above. One mL of hexafluoride lipid-type A microspheres were given per institutional CEUS protocol, which demonstrated a hyperemic gallbladder wall defect in the gallbladder fundus communicating with a fluid-filled, avascular intrabdominal pocket extending into the right pericolic gutter (Figure 3, supplemental video S1). No associated mass or lesion was found within the gallbladder lumen or wall. Therefore, findings were deemed most consistent with primary chronic cholecystitis with echogenic debris within the lumen.

General surgery was consulted in the ED but given her underlying malignancy and metastatic burden, the patient was deemed a poor candidate for operative intervention at the time of presentation. Instead, a 10 French cholecystostomy tube and CT-guided abdominal 12 French catheter were placed into the right abdominal abscess. The patient was admitted and treated with 3.375 g of intravenous piperacillin/tazobactam for 48 hours followed by 4 days of oral amoxicillin/clavulanate. She was discharged in stable condition. Six months later, she underwent delayed cholecystectomy with removal of her drains in the setting of a larger planned procedure for debulking of her ovarian carcinoma by her gynecological surgery team.

## Discussion

Perforation of the gallbladder is a rare and potentially fatal disease, first described and categorized by Niemeier into three types: type I, chronic cholecystoenteric fistula formation; type II, subacute abscess formation; and type III, generalized biliary peritonitis [19]. The present case was consistent with a Niemeier type II perforation. Unlike Niemeier type III perforations, type II perforations do not present with acute peritonitis that indicate emergent operative management but rather with more ambiguity in diagnosis and management. Furthermore, Niemeier type II perforations are the most common of the three, accounting for 46.2-52.6% of all gallbladder perforations [18, 20]. As such, it is important to refine and further develop the tools that may be utilized in raising clinical suspicion and diagnosing this uncommon complication.

While the most common attributed causes of gallbladder perforation are acute cholecystitis and gallstones, several cases of spontaneous gallbladder perforation have been reported in the setting of malignancy and chemotherapy [21–25]. Unfortunately, presenting symptoms vary greatly. While some patients present with fever, nausea with vomiting, and right upper quadrant pain, many patients may present with little to no abdominal pain, as in the present case [26]. CT and ultrasonography have been used to some avail, with the highest sensitivity reported at about 50% [5, 6, 27–33]. Doppler ultrasonography and the use of an UCA may be particularly useful aids in uncertain cases [30, 33]. Unlike conventional US, CEUS shows rapid enhancement of the gallbladder wall during the arterial phase [34]. This allows the operator to better visualize an interruption of the gallbladder wall, which appears as the absence of enhancement of the perforated wall. This can be missed on conventional US which may identify ancillary nonspecific findings such as distension of the gallbladder and gallbladder wall edema, and without wall enhancement, may miss the more specific ‘hole sign’ (defect in the gallbladder wall) [2]. Moreover, CEUS also improves the sensitivity of US in detecting abscess formation in the surrounding liver parenchyma, given earlier enhancement of the gallbladder wall as compared to the surrounding liver parenchyma [34]. Since there is early enhancement of the gallbladder wall in the arterial phase, the integrity of the wall is easily visualized [35]. This is particularly important in cases of impending perforation with areas of wall necrosis but before frank perforation. Given the timely bedside availability of CEUS compared to CT, this could potentially lead to earlier diagnosis before perforation occurs as CEUS can show areas of hypoperfusion with a higher spatial resolution compared to CT. Moreover, given the low sensitivity of both conventional US and CT imaging modalities for detecting gallbladder perforation [5–7], further studies are needed to understand the sensitivity of CEUS in evaluating this pathology. Use of an UCA may also aid in differentiating cases of cholecystitis from gallbladder wall carcinoma that often mimics imaging findings of chronic cholecystitis. The hypo-enhancement time, or contrast agent washout time, may be useful in differentiating between different types of gallbladder lesions; for example, malignant lesions have been noted to have significantly shorter (<50s) washout time when compared to benign lesions [36].

To our knowledge, there is currently sparse literature on the diagnostic usage of CEUS in direct comparison to US, contrast-enhanced CT, or MRI in gallbladder perforation, and no reported studies evaluating point of care CEUS [37]. As this is an uncommon pathology, to the best of our knowledge, this is the first case report on the utility of point of care emergency medicine physician performed CEUS to diagnose gallbladder perforation with pericholecystic abscess. Our work complements a Chinese case series by Tang et al., which provides limited data on six patients with gallbladder perforation for whom experienced US sonographers performed CEUS. Radiology performed CEUS revealed the site of perforation and pericholecystic hepatic abscess in all six cases, whereas only two of the six cases were visualized by conventional B-mode US [38]. A similar case series of eight patients from Italy, with CEUS performed by experienced sonographers, identified a gallbladder wall defect in six patients. This was confirmed as gangrenous/phlegmonous cholecystitis at pathology in all six, and in four as gallbladder perforation during surgical inspection. No studies were identified outside of these small case series [37].

As point of care ultrasonography is now considered standard in emergency medicine , addition of CEUS may aid in the detection of complications of acute or chronic cholecystitis such as gallbladder perforation. This is significant as point of care biliary US conducted by emergency physicians is linked to shorter ED visits and offers access to rapid diagnostic information for bedside clinicians [39]. Moreover, because symptoms and clinical signs of gallbladder perforation can be indistinguishable from those of uncomplicated acute cholecystitis, failure to add CEUS to standard biliary point of care images may lead to delayed or missed diagnosis [40]. Despite these various advantages, when compared to CT, CEUS may be limited by operator proficiency, and this remains a factor for consideration in its diagnostic usage.

In this case, the perforation and abscess formation were clearly visualized. CEUS was able to both confirm perforation and rule out local tumor infiltration, which was later confirmed by pathology after cholecystectomy. The avascular nature of the intrabdominal pocket extending into the right pericolic gutter was less suggestive of malignancy process and was more suggestive of abscess. Here, the identification and confirmation of gallbladder perforation on imaging allowed for more conservative management with image-guided drain placement in a patient who was not an optimal surgical candidate at the time of presentation and underwent delayed cholecystectomy. The standard of care was likely unchanged as the patient was not an optimal surgical candidate at the time of presentation and the perforation and abscess formation were clearly visualized on CT imaging. However, the demonstrated ability of CEUS to detect perforation and provide detailed information about gallbladder wall perfusion and integrity may change management for patients for whom gallbladder perforation was missed on conventional CT or US imaging. Given the low sensitivity of both conventional CT and US imaging modalities for detecting gallbladder perforation [5–7], this case report highlights an area for further research examining the role of CEUS in confirming this diagnosis.

## Conclusions

CEUS should be considered in patients who present with complicated cholecystitis or are at risk for malignancy-related gallbladder perforation and equivocal conventional CT or US imaging. While limited evidence exists to support the use of CEUS at the bedside to make this diagnosis, this case report emphasizes the role of CEUS in providing detailed information about gallbladder wall perfusion and integrity as well as visualizing infiltrative lesions. CEUS may predict impending gallbladder perforation by demonstrating areas of gallbladder wall hypoperfusion and necrosis [41].


